# Whole genome transcriptome data from the WT cortex and hippocampus of female and male control and APP/PS1 Alzheimer's disease mice

**DOI:** 10.1016/j.dib.2023.109594

**Published:** 2023-09-18

**Authors:** Anna Papazoglou, Christina Henseler, Sandra Weickhardt, Johanna Daubner, Teresa Schiffer, Karl Broich, Jürgen Hescheler, Dan Ehninger, Catharina Scholl, Britta Haenisch, Agapios Sachinidis, Marco Weiergräber

**Affiliations:** a Experimental Neuropsychopharmacology, Federal Institute for Drugs and Medical Devices (Bundesinstitut für Arzneimittel und Medizinprodukte, BfArM), Kurt Georg-Kiesinger-Allee 3, 53175 Bonn, Germany; b Federal Institute for Drugs and Medical Devices (Bundesinstitut für Arzneimittel und Medizinprodukte, BfArM), Kurt-Georg-Kiesinger-Allee 3, 53175 Bonn, Germany; c Faculty of Medicine, Institute of Neurophysiology, University of Cologne, Robert-Koch-Str. 39, 50931 Cologne, Germany; d Center of Physiology and Pathophysiology, Faculty of Medicine, University of Cologne, Robert-Koch-Str. 39, 50931 Cologne, Germany; e Translational Biogerontology, German Center for Neurodegenerative Diseases (Deutsches Zentrum für Neurodegenerative Erkrankungen, DZNE), Venusberg-Campus 1/99, 53127 Bonn, Germany; f German Center for Neurodegenerative Diseases (Deutsches Zentrum für Neurodegenerative Erkrankungen, DZNE), Venusberg-Campus 1/99, 53127 Bonn, Germany; g Center for Translational Medicine, Medical Faculty, University of Bonn, 53113 Bonn, Germany

**Keywords:** Amyloid precursor protein, Brain, Hippocampus, Hybridization, Microarray, Retrosplenial (RS) cortex, RNA, Transcriptome

## Abstract

A variety of Alzheimer disease (AD) mouse models has been established and characterized within the last decades. These models are generated to meet the principal criteria of AD isomorphism, homology and predictability to a maximum extent. To get an integrative view of the sophisticated etiopathogenesis of AD, whole genome transcriptome data analysis turns out to be indispensable. Here, we present a microarray-based transcriptome data collection based on RNA extracted from the retrosplenial (RS) cortex and the hippocampus of APP/PS1 AD mice and control animals. Experimental animals were age matched and importantly, both sexes were considered separately. Isolated RNA was purified, quantified und quality controlled prior to the hybridization procedure with SurePrint G3 Mouse Gene Expression v2 8 × 60K microarrays. Following immunofluorescent measurement und preprocessing/extraction of image data, raw transcriptome data were uploaded including differentially expressed gene candidates and related fold changes in APP/PS1 AD mice and controls. Our data allow further insight into alterations in gene transcript levels in APP/PS1 AD mice compared to controls and enable the reader/user to carry out complex transcriptome analysis to characterize potential age-, sex- and brain-region-specific alterations in e.g., neuroinflammatory, immunological, neurodegenerative and ion channel pathways.

Specifications Table

**Table:** 

Subject	Biology
Specific subject area	Murine cortical and hippocampal transcriptomics
Type of data	Tables, images, transcriptome data (raw csv.-files)
How the data were acquired	Transcriptome data were acquired using the One-Color Microarray-Based Gene Expression system by Agilent Technologies Germany GmbH & Co.KG, Germany. In specific, the SurePrint G3 Mouse Gene Expression v2 8 × 60K Microarray Kit (Agilent Technologies Germany GmbH & Co. KG, Germany) was used for RS cortex and hippocampal tissue. All procedures were carried out according to the manufacturer's instructions (see below). The raw data are based on fluorescence scanning using the Agilent SureScan Microarray Scanner and raw microarray image file processing using the Feature Extraction Software (both Agilent Technologies Germany GmbH & Co. KG, Germany). Using GeneSpring Software (Agilent Technologies Germany GmbH & Co. KG, Germany), all information about differentially expressed genes, their fold changes, statistics etc. were extracted.
Data format	Raw and filtered/pre-analyzed data (txt./csv.-files) The txt./csv.-files allow usage in other transcriptome analysis software in case GeneSpring software is not used and/or available.
Description of data collection	Total RNA from the RS cortex and hippocampus was extracted from age-matched female and male control and APP/PS1 AD mice (8 control animals (4 ♂, age: 32.72 ± 0.38 weeks; 4 ♀, age: 32.14 ± 0.25 weeks) and 8 APPswePS1dE (APP/PS1) AD mice (3 ♂, age: 32.81 ± 0.24 weeks; 5 ♀, age: 32.66 ± 0.39 weeks). Isolated RNA was hybridized to the SurePrint G3 Mouse Gene Expression v2 8 × 60K Microarrays to unravel transcriptional alterations in APP/PS1 AD mice compared to wild-type (WT) control animals.
Data source location	Federal Institute for Drugs and Medical Devices (Bundesinstitut für Arzneimittel und Medizinprodukte, BfArM) Kurt-Georg-Kiesinger Allee 3 53175 Bonn Germany
Data accessibility	Data is available at MENDELEY DATA (doi:10.17632/z9264694b4.2) for WT and for APP/PS1 AD mice. Repository name: MENDELEY DATA Data identification number: doi:10.17632/z9264694b4.2 Direct URL to data: https://data.mendeley.com/drafts/z9264694b4

## 1 Value of the Data

• These data provide a basis for the analysis of candidate genes that might be important in immunological and inflammatory responses, synaptic integration, learning and memory and ictogenesis in AD.
• Researchers in preclinical and clinical AD studies can benefit from these data for characterizing new sex- and brain-region specific candidate genes that might play a role as prospective or disease progression markers.
• These data can further be used for translational studies and serve as a gateway to precision medicine, AD patient stratification and personalized treatment.
• Our sex- and brain region-specific transcriptome data allow for further investigation of differentially expressed, intersectional and signature genes, gene ontology/enrichment analysis and pathway studies.
• Our data can serve as a benchmark AD transcriptome dataset that allows comparison with human transcriptome data and other rodent AD lines with different age, sex and brain regions of interest.

## 2 Objective

In this study, the double transgenic APPswePS1dE (APP/PS1) AD mouse model has been utilized. Both mutations are associated with early-onset AD [1], [2], [3], [4]. The first Aβ plaques in this line can be detected at around 4 months of age, mainly in the cortex and hippocampus. At the age of 5 to 12 months, mice start displaying sex-specific differences in Aβ deposition in the brain [5]. Furthermore, progressive behavioral and cognitive deficits become obvious in spatial navigation, reference learning, and Morris water maze [6]. In addition, seizure activity is often observed in APP/PS1 AD mice and might be responsible for sudden death in this line as well [7,8]. To understand the numerous pathophysiological alterations in APP/PS1 AD mice, a detailed investigation of genome-wide transcriptional alterations is indispensable. Importantly, our transcriptome data can provide potential mechanistic information about metabolic/biochemical, signal transduction, immunological, inflammatory, neurodegenerative and electrophysiological implications in APP/PS1 AD mice in an age-, sex- and brain-region-specific manner. In particular, the sex-specific differences in behavioral and cognitive decline in AD have gained more and more attention [9,10].

## 3 Data Description

**Fig. 1:**
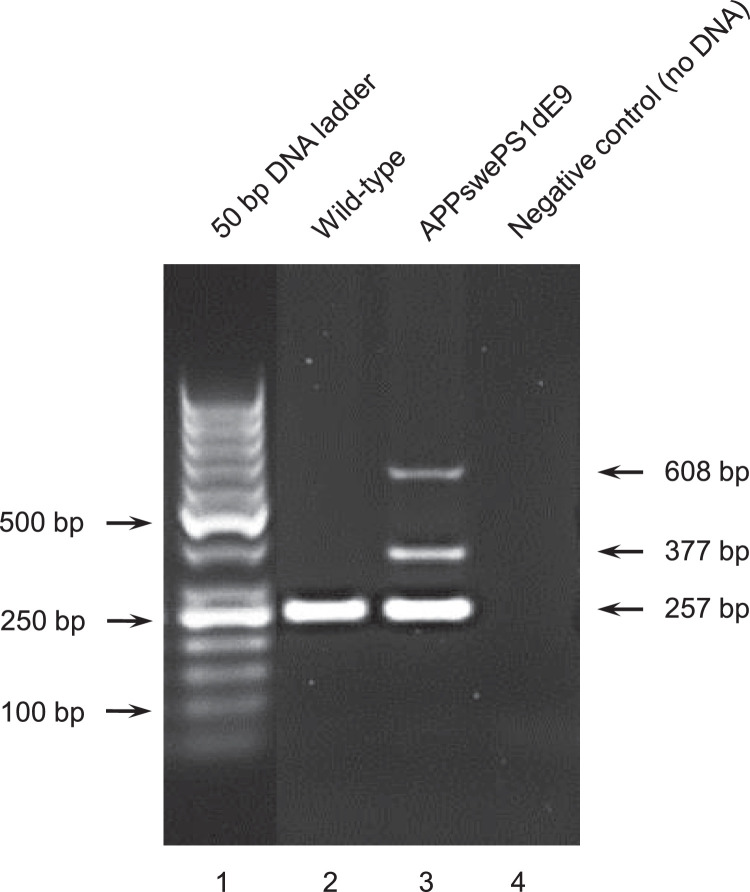
Representative genotyping image of an APPswePS1dE9 (APP/PS1) AD mouse (lane 3) and a WT control mouse (lane 2). Individual genotypes are characterized by specific DNA fragments. The 377 bp fragment is indicative of the mutant APP variant, whereas the 608 bp fragment indicates the mutant PS1 variant. The muscarinic acetylcholine receptor 5 (Chrm5) was used as a positive WT control (see 257 bp fragment). For the negative control (no genomic DNA), see lane 4.

**Fig. 2:**
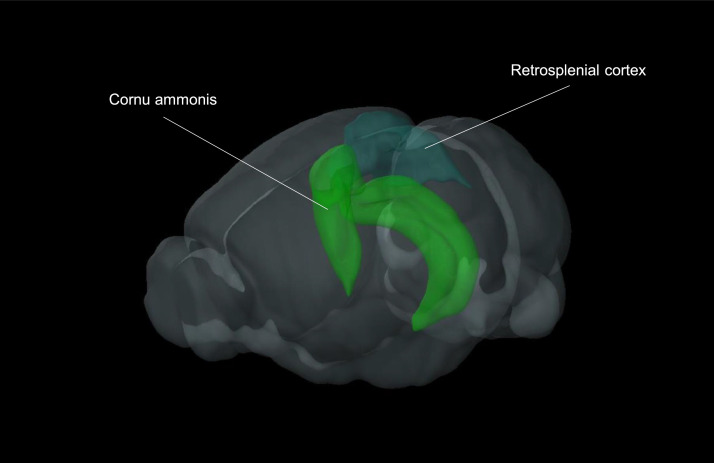
3D view of the mouse brain and extirpated brain regions of interest. The RS cortex and cornu ammonis are highlighted. Both regions were extirpated and subsequently used for RNA isolation (The 3D image was created using the Allen Brain Explorer^Ⓡ^ beta version (https://connectivity.brain-map.org/static/brainexplorer) [11].

Retrosplenial cortex and hippocampus were isolated from 8 months old controls and APP/PS1 AD mice from both sexes (Figs. 1, 2). Following cortical and hippocampal RNA isolation, microarray procedures were carried out to acquire the transcriptome profile of the animals under investigation (see Table 1, Table 2, Table 3). The raw reads are accessible at MENDELEY DATA (doi:10.17632/z9264694b4.2) for control animals and for APP/PS1 AD mice.

**Table 1:** Composition of PCR reaction mix used for genotyping.

Reagent	Volume per reaction (µl)
ddH_2_O	8.7
Forward Primer APP (25 µM)	0.3
Reverse Primer APP (25 µM)	0.3
Forward Primer PS1 (25 µM)	0.3
Reverse Primer PS1 (25 µM)	0.3
Forward Primer WT control (25 µM)	0.3
Reverse Primer WT control (25 µM)	0.3
Red Taq Ready Master Mix	12.5
genomic DNA	2
*Total volume*	*25*

**Table 2:** Material (kits) used for one-color microarray-based gene expression data collection.

Kit	Catalog number	Company
Agilent RNA 6000 Nano Kit	5067-1511	Agilent Technologies Germany GmbH & Co. KG, Waldbronn, Germany
Gene Expression Hybridization Kit	5188-5242	Agilent Technologies Germany GmbH & Co. KG, Waldbronn, Germany
Gene Expression Wash Buffer Kit	5188-5327	Agilent Technologies Germany GmbH & Co. KG, Waldbronn, Germany
Hybridization Gasket Slide Kit (20)	G2534-60015	Agilent Technologies Germany GmbH & Co. KG, Waldbronn, Germany
Low Input Quick Amp Labeling Kit, One-Color	5190-2305	Agilent Technologies Germany GmbH & Co. KG, Waldbronn, Germany
One-Color RNA Spike-In Kit	5188-5282	Agilent Technologies Germany GmbH & Co. KG, Waldbronn, Germany
RNase-free DNase Set (50)	79254	Qiagen GmbH, Hilden, Germany
RNeasy Lipid Tissue Mini Kit (50)	74804	Qiagen GmbH, Hilden, Germany
RNeasy Mini Kit	74104	Qiagen GmbH, Hilden, Germany
SurePrint G3 Mouse Gene Expression v2 8 × 60K Microarray Kit	G4852B	Agilent Technologies Germany GmbH & Co. KG, Waldbronn, Germany

**Table 3:** Software used for one-color microarray-based gene expression data collection and extraction.

Software	Version	Company
2100 Expert Software	B.02.08.SI648 (SR1)	Agilent Technologies Germany GmbH & Co. KG, Waldbronn, Germany
Feature Extraction Software	11.01.1	Agilent Technologies Germany GmbH & Co. KG, Waldbronn, Germany
GeneSpring GX	14.9.1	Agilent Technologies Germany GmbH & Co. KG, Waldbronn, Germany
Microarray Scan Control Software	9.1.11.7	Agilent Technologies Germany GmbH & Co. KG, Waldbronn, Germany
NanoDrop ND-1000	3.8.1	Thermo Fisher Scientific Inc., USA

Note that the transcriptome data of the experimental animals were labeled as follows in the Mendeley database:

- Four male control mice: Control #1, Control #2, Control #3, Control #4 (note that for control #4 no transcriptome data from the RS cortex were obtained)
- Four female control mice: Control #5, Control #6, Control #7, Control #8
- Three male APP/PS1 AD mice: APP/PS1 #1, APP/PS1 #2, APP/PS1 #3
- Five female APP/PS1 AD mice: APP/PS1 #4, APP/PS1 #5, APP/PS1 #6, APP/PS1 #7, APP/PS1 #8 (note that for APP/PS1 #8 no transcriptome data from the hippocampus were obtained)

## 4 Experimental Design, Materials and Methods

### 4.1 Experimental animals

Transcriptome data were obtained from the double transgenic APPswePS1dE9 (APP/PS1) AD mice with a C57BL/6J background. This AD mouse line carries a chimeric mouse/human amyloid precursor protein (APP) with two Swedish mutations (APPswe) co-integrated with human Presenilin 1 (PS1) with exon 9 deletion (PS1-dE) [1,3,4]. Mutant mice (B6.Cg-Tg(APPswe, PSEN1dE9)85Dbo/Mmjax, MMRRC stock no. 34832-JAX) and their WT littermates were purchased from the Jackson Laboratory (USA). In total, 8 control animals (4 ♂, age: 32.72 ± 0.38 weeks; 4 ♀, age: 32.14 ± 0.25 weeks) and 8 APPswePS1dE (APP/PS1) AD mice (3 ♂, age: 32.81 ± 0.24 weeks; 5 ♀, age: 32.66 ± 0.39 weeks) were used for hippocampal and cortical extirpation and subsequent transcriptome analysis. Importantly, both sexes were used separately. Note that no information about the stage of the estrous cycle of the female experimental animals is available.

All experimental mice were housed in groups of 3-4 in clear Makrolon cages type II with *ad libitum* access to drinking water and standard food pellets. Mice were maintained inside ventilated cabinets (Type Uniprotect, Zoonlab, Germany) at an ambient temperature of 21 ± 2°C, 50–60% relative humidity, and on a conventional 12 h/12 h light/dark cycle beginning at 5:00 am. All animals were strictly adapted to the circadian pattern preceding cortical and hippocampal extirpation and RNA isolation (see below).

### 4.2 Genotyping - DNA preparation from tail biopsies

Every experimental animal was genotyped twice using DNA isolated from tail biopsies. DNA preparation was carried out using peqGOLD DNA Mini Kit (PEQLAB Biotechnologie GmbH, Germany) according to the manufacturer's instructions. The isolated genomic DNA was stored at +4°C until further use.

### 4.3 Genotyping - polymerase chain reaction (PCR)

For every sample, a PCR reaction mix containing three different primer pairs (APP forward: 5’-AGGACTGACCACTCGACCAG-3’; APP reverse: 5’-CGGGGGTCTAGTTCTGC-3’; PS1 forward: 5’-AATAGAGAACGGCAGGAGCA-3’; PS1 reverse: 5’-GCCATGAGGGCACTAATCAT-3’; WT control forward (muscarinic receptor 5, Chrm5) 5’- ACCTTGGACCAAATCTGAGTGTA-3’; WT control reverse (muscarinic receptor 5, Chrm5): 5’- GGCCAAGCTGAGCAGGTAAT-3’), ddH_2_O (PCR grade), Red Taq Ready Master Mix (Sigma Aldrich, Germany) and isolated sample specific genomic DNA (∼20 ng/µl) was prepared (Table 1).

The PCR reaction mix was gently vortexed for 3 sec followed by a brief centrifugation step for 5 sec at 2000xg using a micro centrifuge (ROTILABO, Carl Roth, Germany). For each genotyping PCR, transgenic DNA, WT DNA (as positive control) and a negative control (no DNA) were added for experimental validation. PCR was carried out using a Bio-Rad C1000 thermal cycler (Bio-Rad Laboratories GmbH, Germany) and the following amplification parameters were applied:

- 94°C, 3 min pre-incubation
- 35 cycles: 94°C, 30 sec denaturation; 48°C, 30 sec annealing; 72°C, 1 min extension
- 72°C, 10 min
- Storage at 4°C till further use

PCR products were analyzed by horizontal agarose gel (1.5% in 0.5x TBE buffer (pH8)) electrophoresis and visualized by ethidium bromide (0.3 µg/ml). The ChemiDoc Touch System (Bio-Rad Laboratories GmbH, Germany) was used for gel imaging and identification of amplified DNA fragments (Fig. 1).

### 4.4 Retrosplenial cortex and hippocampus preparation and tissue storage

Experimental animals were deeply anaesthetized using i.p. injection of ketamine (100 mg/kg) / xylazine (10 mg/kg). To ensure that the animals were fully anaesthetized, the absence of tail and foot pinch reflexes was verified. Animals were decapitated, the brain was immediately removed and placed in a clean RNase-free petri-dish on ice filled with pure RNAlater reagent (Qiagen GmbH, Germany). By using a scalpel, forceps and a thin brush, the whole hippocampus and a piece (2-3 mm^3^) of the RS cortex were dissected from both brain hemispheres (Fig. 2). Each tissue fragment was placed in a 2 ml RNase free reaction tube, snap frozen in liquid nitrogen and stored at -80°C until RNA isolation. Note that to avoid potential interference of transcriptional profiles and circadian rhythmicity, tissue preparation was always carried out ante meridiem between 8 am and 11 am.

### 4.5 Cortical and hippocampal RNA isolation

Total RNA was isolated using RNeasy Lipid Tissue Mini Kit (Qiagen GmbH, Germany) according to the manufacturer's instructions (incl. optional DNase digestion step). Cortical and hippocampal tissue samples were removed from -80°C, immediately lysed in QIAzol lysis buffer (Qiagen GmbH, Germany) and homogenized using the TissueRuptor^Ⓡ^ (Qiagen GmbH, Germany), a handheld-rotor-stator homogenizer with disposable probes. Following phenol-chloroform separation, DNase digestion and three washing steps, total RNA was eluted in 30 µl RNase-free ddH_2_O. Isolated RNA for transcriptome studies was measured using NanoDrop^Ⓡ^ ND-1000 (Thermo Fisher Scientific, USA) according to the manufacturer's instructions. The ratio of absorbance at 260 and 280 nm (260/280) was used to assess RNA purity with a ratio of ∼ 2.0 being generally accepted as “pure” for RNA related experimental approaches (see NanoDrop^Ⓡ^ user manual). The ratio of absorbance at 260 and 230 nm (260/230) is a secondary measure of nucleic acid purity. They are commonly in the range of 1.8-2.2 (see NanoDrop^Ⓡ^ user manual). Our RNA probes from APP/PS1 and control BROI samples for microarray experiments exhibited absorbance ratios of ∼ 2.0 (for 260 nm/280 nm) and of 2.05 (for 260 nm/230 nm).

### 4.6 One-color microarray-based gene expression analysis

The One-Color Microarray-Based Gene Expression system by Agilent Technologies Germany GmbH & Co. KG (Germany) utilizes the Low Input Quick Amp Labeling Kit to generate cDNA (1^st^ and 2^nd^ strand cDNA) via AffinityScript-RT (reverse transcriptase) which is a genetically engineered, highly thermostable version of MMLV (Moloney Murine Leukemia Virus enzyme) RT (Table 2). Subsequently, the samples were labeled with a T7 RNA polymerase blend. This polymerase incorporates Cyanine 3-CTP during amplification generating an one-color fluorescent complimentary RNA (cRNA) as target material. The RNA sample input is supposed to range from 10 - 200 ng (for details on the procedure see manufacturer's instructions in the One-Color RNA Spike-In Kit and Low Input Quick Amp Labeling Kit, (both from Agilent Technologies Germany GmbH & Co. KG, Germany) (Table 2)). The amplification from total RNA to amplified cRNA is typically around 100-fold. Subsequently, the labeled/amplified cRNA is purified using the RNeasy Mini Kit (Qiagen GmbH, Germany) followed by quantification of the cRNA using the NanoDrop ND-1000 UV-VIS spectrophotometer (ThermoFisher Scientific Inc., USA). After finalization of cRNA sample preparation, hybridization of samples is carried out for 17 hrs (65°C) using the Gene Expression Hybridization Kit and the SurePrint G3 Mouse Gene Expression v2 8 × 60K Microarray Kit (both from Agilent Technologies Germany GmbH & Co. KG, Germany) according to the manufacturer's instructions (Table 2). After the washing procedure with Gene Expression Wash Buffer Kit (Agilent Technologies Germany GmbH & Co. KG, Germany) to remove unspecific bindings, the microarrays are prepared for scanning and feature extraction. The microarray scan was performed using the Agilent SureScan Microarray Scanner (Agilent Technologies Germany GmbH & Co. KG, Germany). Feature Extraction Software (Agilent Technologies Germany GmbH & Co. KG, Germany) was used to extract the information from the probe features of the microarray scan data, providing information about gene expression/transcripts for further analysis (Table 3).

## Ethics Statement

All animal procedures were carried out in accordance with the Guidelines of the German Council on Animal Care and all protocols were approved by the Local Institutional and National Committee on Animal Care (approval number AZ84-02.04.2013.A426; Landesamt für Natur, Umwelt und Verbraucherschutz, LANUV, Germany). The authors further certify that all animal experimentation complied with the ARRIVE guidelines and were carried out in accordance with the U.K. Animals (Scientific Procedures) Act, 1986 and associated guidelines; EU Directive 2010/63/EU for animal experiments; or the National Institutes of Health guide for the care and use of laboratory animals (NIH Publications No. 8023, revised 1978). Maximum effort was made to reduce the number of animals necessary to obtain data and suffering of the animals according to the 3R strategy.

## CRediT authorship contribution statement

**Anna Papazoglou:** Conceptualization, Methodology, Visualization, Investigation, Software, Validation, Writing – review & editing. **Christina Henseler:** Conceptualization, Methodology, Software, Visualization, Investigation, Validation, Writing – review & editing. **Sandra Weickhardt:** Writing – review & editing. **Johanna Daubner:** Conceptualization, Methodology, Software, Validation, Writing – review & editing. **Teresa Schiffer:** Conceptualization, Methodology, Software, Validation. **Karl Broich:** Writing – review & editing. **Jürgen Hescheler:** Writing – review & editing. **Dan Ehninger:** Writing – review & editing. **Catharina Scholl:** Writing – review & editing. **Britta Haenisch:** Writing – review & editing. **Agapios Sachinidis:** Conceptualization, Methodology, Software, Writing – review & editing. **Marco Weiergräber:** Data curation, Writing – original draft, Visualization, Investigation, Supervision, Software, Validation.

## Data Availability

APP/PS1 (Original data) (Mendeley Data).
